# Machine learning methods trained on simple models can predict critical transitions in complex natural systems

**DOI:** 10.1098/rsos.211475

**Published:** 2022-02-16

**Authors:** Smita Deb, Sahil Sidheekh, Christopher F. Clements, Narayanan C. Krishnan, Partha S. Dutta

**Affiliations:** ^1^ Department of Mathematics, Indian Institute of Technology Ropar, Rupnagar, Punjab 140001, India; ^2^ Department of Computer Science and Engineering, Indian Institute of Technology Ropar, Rupnagar, Punjab 140001, India; ^3^ School of Biological Sciences, University of Bristol, Bristol BS8 1TQ, UK

**Keywords:** catastrophic transitions, non-catastrophic transitions, tipping points, early warning indicators, classification, deep learning

## Abstract

Forecasting sudden changes in complex systems is a critical but challenging task, with previously developed methods varying widely in their reliability. Here we develop a novel detection method, using simple theoretical models to train a deep neural network to detect critical transitions—the Early Warning Signal Network (EWSNet). We then demonstrate that this network, trained on simulated data, can reliably predict observed real-world transitions in systems ranging from rapid climatic change to the collapse of ecological populations. Importantly, our model appears to capture latent properties in time series missed by previous warning signals approaches, allowing us to not only detect if a transition is approaching, but critically whether the collapse will be catastrophic or non-catastrophic. These novel properties mean EWSNet has the potential to serve as an indicator of transitions across a broad spectrum of complex systems, without requiring information on the structure of the system being monitored. Our work highlights the practicality of deep learning for addressing further questions pertaining to ecosystem collapse and has much broader management implications.

## 1. Introduction

Transitions from one steady state to another occur in many complex systems, such as financial markets [1], human societies [2–4], climate systems [5–7], systems biology [8] and ecosystems [9–11]. Such transitions can be catastrophic (i.e. sudden, large and often irreversible changes in the state of a system), or non-catastrophic (i.e. smooth and reversible, and characterized by quantitatively similar dynamics prior to and post transition), and can occur due to gradual external forcing or random fluctuations in the system. In such scenarios, on crossing a threshold (known as a tipping or bifurcation point), structural changes occur in the underlying system. This is often termed a critical transition, prior to which the system’s return to an equilibrium slows down—a phenomenon known as critical slowing down (CSD) [3]. The phenomenon of CSD is related to the fact that the real part of the dominant eigenvalue of the system goes to zero at the bifurcation point [12,13]. In all such cases, where the dominant eigenvalue approaches zero close to the tipping point irrespective of catastrophic or non-catastrophic transitions, the phenomenon of CSD persists, and there exist statistical indicators that forewarn the vicinity of a tipping point [14]. Understanding the causes of sudden transitions and forecasting them using statistical indicators have recently emerged as an important area of research due to the management implications of preventing catastrophes in natural systems [12,15,16].

The traditional approach of forecasting a critical transition relies on summary statistics such as variance, autocorrelation and skewness showing an increasing trend before a transition. These traditional CSD-based early warning signals (EWSs) are generic to any phenomena where the dominant eigenvalue of the system’s Jacobian matrix tends to zero. This means that such EWSs are applicable to a vast array of systems, but their robustness in forecasting critical transitions remains debatable [17–20]. The uncertainties in generic EWSs can be attributed to factors including imperfect data sampling, lack of quantitative and objective measures, short time series, and sensitivity to bandwidth and window sizes. However, despite these challenges, significant work has sought to increase the statistical power of the EWSs in the hope they can be used as predictive management tools. This includes recent work showing that incorporating additional measures of the health of a system (such as the mean body size of biological populations) can increase the efficiency in predicting a tipping point [21–23]. While such approaches are promising, they require more data than traditional generic EWSs (two or more simultaneous time series), limiting their applicability to many systems [21]. Consequently, there remains a critical need to develop a robust toolkit to identify tipping points using widely available time-series data. Were this to be achieved, it could have significant implications for the management of a host of systems, from financial markets to species at risk of extinction [8,10,11].

One potentially powerful tool to achieve this is machine learning (ML). ML models are able to automatically capture statistical characteristics by identifying and learning patterns in data [24], making them ideally suited to detecting warning signals (see electronic supplementary material, appendix, section S1). While a caveat of ML models is their applicability to data following similar distribution as that of training data (i.e. if the model is trained on cyclical data then the test data should also be cyclical), they are now widely used in a number of disciplines including atmospheric science (weather forecast) [25], economics (finance) [26], biological sciences (medical diagnosis) [27], physical (quantum systems) [28] and mathematical sciences (nonlinear dynamics) [24]. Indeed, in fields relating to critical transitions, ML has been used to classify phases of matter, study phase behaviour, detect phase transitions, and predict chaotic dynamics [24,29–32], while supervised learning algorithms such as artificial neural networks have been used to study the second-order phase transitions, especially the Ising model [33–36]. However, thus far ML tools have not been used to classify the most common transitions seen in ecological, financial and climatic systems—catastrophic (i.e. first order or discontinuous) and non-catastrophic (i.e. second order or continuous) transitions [37].

In this study, we propose an Early Warning Signal Network (EWSNet) framework for predicting transitions. EWSNet is a parametrized function deploying long short-term memory (LSTM) and fully convoluted network (FCN) sub-modules. The LSTM sub-module is capable of processing sequential data (such as text, audio, video, etc.) and captures long-term dependencies in the time series [38]. The FCN sub-module extracts complex nonlinear patterns from the data [39]. The sub-modules together learn the characteristics indicative of an impending transition. EWSNet is trained on time-series data simulated from nine different dynamical models, including biological, ecological and climate models displaying catastrophic, non-catastrophic and no transitions (see electronic supplementary material, appendix, section S2 and table S1), and then validated on time series from the above models. We show that EWSNet provides robust identification of approaching tipping points in simulated data, and that it outperforms the four classical ML models (logistic regression, support vector machine (SVM), random forest and multilayer perceptron (MLP) [40–42]) which are trained to classify time series based on trends in their statistical properties such as autocorrelation—the basis for generic early warning signals [15]. Furthermore, we then show that, even though EWSNet is trained on simulated time-series data, it can classify approaching transitions in real-world and experimental datasets [5,21,22,43] with high prediction probability suggesting further investigation of the data. Our results suggest that EWSNet can reliably predict the future state of a range of complex systems even when time series are imperfectly sampled [19]. The approach of the EWSNet as an early warning indicator makes an assumption about the underlying state of the system: that the test data should follow a distribution similar to the training data. It requires no data pre-processing, and is invariant to sequence length, and thus offers a novel predictive management tool.

## 2. Models and methods

### 2.1. Simulated training data

We have generated stochastic time series from nine different models ranging from ecological to palaeoclimatic systems that cover a wide range of nonlinearities (see electronic supplementary material, appendix, section S2). The considered models are of the form:

2.1 $$\frac{dX}{dt}=f\;(X)+g(X)\xi (t),$$

where *X* is the state variable, *f*(*X*) is the deterministic skeleton of the model, *g*(*X*) is an arbitrary function and *ξ*(*t*) is a random variable depicting coloured noise. The effect of coloured noise was incorporated in the deterministic skeleton by the equation

2.2 $$\xi (T+1)=k\xi (T)+\sigma \sqrt{1-k^{2}}\frac{\phi (T)+\beta w(T)}{\sqrt{1-\beta^{2}}},$$

where $\beta =\sqrt{(1-|\rho |)/\left|\rho \right|}$, *ρ* is the species response correlation, *T* represents the time points (1, …, 400), *k* is the autocorrelation coefficient, and *σ* is the noise intensity. *ϕ*(*T*) and *w*(*T*) are normal random components, where *w*(*T*) differs across species unlike *ϕ*(*T*) [44,45]. The stochastic models were simulated using the Euler–Maruyama method with *k* ∈ [ − 0.8, 0.8]. We majorly trained our deep neural network using a large number of simulated time-series data perturbed with white noise (i.e. *k* = 0). For testing our model potency in anticipating transitions in time series for systems perturbed with multiplicative noise, we trained EWSNet with an additional number of time series which have been perturbed with coloured noise. This is done in order to let the EWSNet be familiar with the fast-changing dynamics and short-scale fluctuations [20] that occur due to the presence of multiplicative noise, and also to test the skills of EWSNet in testing datasets which may not fall in the known regimes of training data.

### 2.2. Deep learning model: EWSNet structure

**Figure 1.**
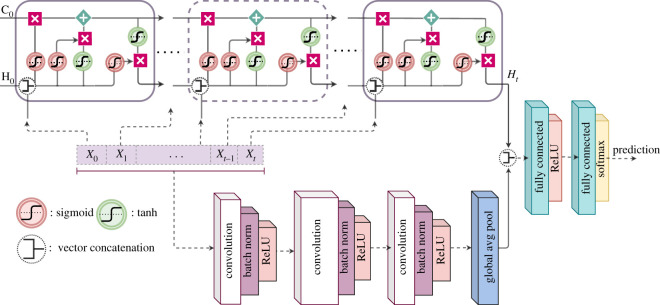
Schematic of the EWSNet: The EWSNet consists of three convolution blocks and an LSTM block. The fully convoluted network and the LSTM block process the input sequence independently. The concatenated output of the two blocks is passed through two fully connected layers to obtain the final prediction. *X*_*t*_ represents the input at time step *t*. C_0_ and H_0_ represent the initial cell and hidden states of the LSTM block, respectively. The cell state allows the passage of stored information, and the hidden state acts as the working memory to the LSTM block of the EWSNet. The global average pooling layer at the end of the convolutional block makes the EWSNet invariant to sequence length.

The EWSNet (figure 1) can be viewed as a large composition of complex nonlinear functions that learn hierarchical representations of the data. The input to the EWSNet is a univariate time series signal. The EWSNet comprises FCN and LSTM blocks (electronic supplementary material, appendix, section S1), followed by fully connected layers [46]. The FCN consists of three stacked convolutional blocks, each composed of convolution [47], batch normalization (BN) [48] and rectified linear unit (ReLU) [49] activation layers. The convolution operation is performed using a filter $W\in R^{1\times k}$ over an input tensor $X\in R^{1\times T}$, where *T* is the length of the time series. These filters are learnable and often characterize various local patterns present in the input tensor. The convolution operation is followed by batch normalization to remove the covariate shift in the output across different training batches. The ReLU activation function is applied to the batch-normalized output. The resulting output at the end of one convolution block can be represented as

2.3 Z=ReLU(BN(W ∗ X)),

where ∗ represents the convolution operation. Each of the three convolution blocks of EWSNet processes the output of the previous block in a similar fashion. The output of the third convolutional block, containing *D* filters, is a set of *D* vectors, each of length *T*. To make EWSNet invariant to the sequence length *T*, we apply a global average pooling operation (over *T*) to obtain a *D*-dimensional vector. Choice of hyperparameters; such as number of filters in convolution blocks and hidden state units in LSTM block are obtained after fine-tuning the deep learning model (electronic supplementary material, appendix, section S1, figure S1).

More details about EWSNet and a user’s guide can be found in the website: https://ewsnet.github.io/.

### 2.3. Machine learning models trained on generic EWSs

We use classical ML models, namely logistic regression, random forest, SVM, MLP, to classify time series based on the extracted EWSs passed as input to these models. Generic EWSs are calculated using time series for a combination of bandwidths ({20, 30, 40}) and window sizes ({40, 50, 60}). This is done with the idea that the EWSs for a particular window size and bandwidth capture certain characteristic features that aid in classifying the time series and assigning the appropriate label (see electronic supplementary material, appendix, section S3). Accordingly, if the individual EWSs for the combinations are concatenated and passed as input to these models, they act as an additional filter to the generic EWSs and are supposed to improve them. A brief description of the model along with the tuned hyperparameters for the respective models are discussed in electronic supplementary material, appendix, section S3, table S2.

## 3. Results

Our deep learning model—EWSNet has been trained to infer approaching transitions from features present in time-series data. The EWSNet (figure 1) consists of two blocks (two independent branches); the LSTM block composed of 128 LSTM (hidden state) units that captures the latent temporal properties and the fully convolutional block that views the time series as spatial data. The hyper-parameters of the model (such as the number of convolution blocks, LSTM hidden units, learning rate, etc.) were carefully fine-tuned using the training and the validation sets (see Models and methods). Two experimental regimes derived from nine different models pertaining to biological, ecological and palaeoclimatic dynamics [5,16,50–52] (see electronic supplementary material, appendix, table S1) perturbed with white noise (Dataset-W) and coloured noise (Dataset-C) are used to train and test the performance of EWSNet. The time series exhibit catastrophic and non-catastrophic transitions with CSD (spanning over four different bifurcations; viz, saddle-node (fold), transcritical, pitchfork and supercritical Hopf bifurcations) and no transitions. We have also included in the study time series with coloured noise where the generic EWSs typically show weak trends [51,53,54]. To estimate the robustness of the models and to rule out results due to chance, we report the performance of the models averaged over 25 trials.

### 3.1. Detecting and characterizing transitions using EWSNet

**Figure 2.**
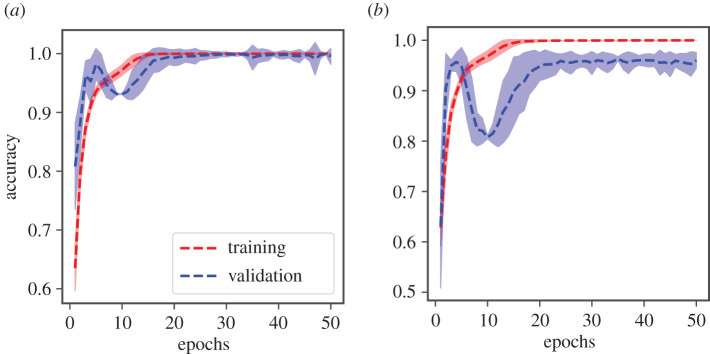
Mean accuracy of EWSNet: for (*a*) Dataset-W, and (*b*) Dataset-C. The mean training and validation accuracies are computed after every epoch and are averaged over 25 trials. The shaded regions represent 95% confidence interval. The accuracies saturate after around 25 epochs indicating the models’ convergence. Accuracy is reported after validating on EWSNet composed of three fully convoluted blocks and an LSTM block with 128 LSTM units. Unless stated, this is the same for all other figures.

**Figure 3.**
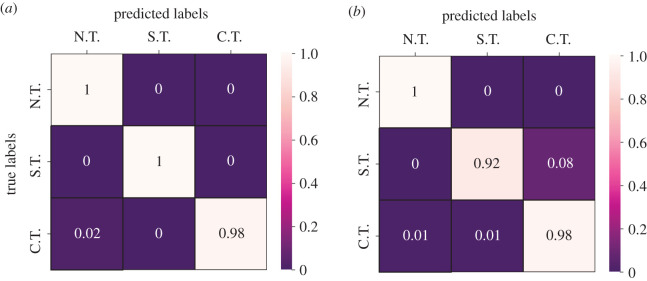
Error analysis using the confusion matrix: for (*a*) Dataset-W, and (*b*) Dataset-C. The EWSNet always classifies a no-transition time series accurately. It rarely makes an error in classifying catastrophic and non-catastrophic transitions for Dataset-W. For Dataset-C, 1% of catastrophic transitions are labelled as non-catastrophic, and 8% of non-catastrophic transitions are being misclassified as catastrophic. Here, C.T., S.T. and N.T. stand for a catastrophic, non-catastrophic/smooth and no transition, respectively.

Two different EWSNet models were each trained independently on Dataset-W and Dataset-C, respectively, using 80% of the generated dataset. The remaining 20% was used for testing the performance of the trained model. Further, the test set of Dataset-C contained time series with highly correlated noise, while the training set contained time series with weakly correlated noise. The training and validation accuracies for both the models as a function of the number of training epochs averaged over 25 different trials are presented in figure 2. We observe the convergence of the model after 25 epochs, as well as transients in the training epochs that are symptomatic of training deep neural network models. The mean test accuracy of the EWSNet models for Dataset-W is 99.46% and for Dataset-C is 95.93%. As can be observed from figure 3, the EWSNet models show high accuracy for all the three labels (catastrophic (C.T.), non-catastrophic/smooth (S.T.) and no transition (N.T.)) on both the datasets. While non-transition series are always correctly classified by both the models, there exists some misclassifications for the other two classes. Moreover, the efficacy of EWSNet for time series of varying lengths and at varying distances from the tipping point (if present) are discussed in the supplementary material (see electronic supplementary material, appendix, section S2, figure S2). The generic EWSs are sensitive to the length of pre-transition time series used, whereas the EWSNet models are marginally affected by reduced time series length.

**Figure 4.**
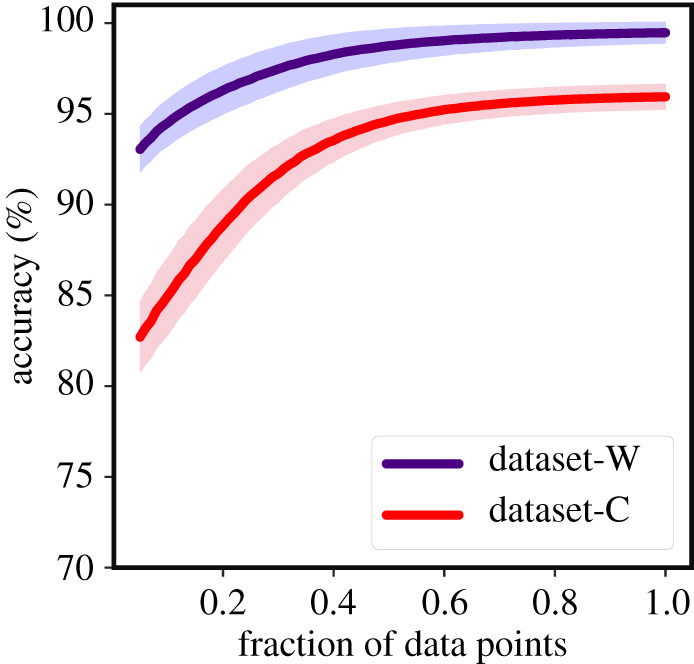
Robustness of EWSNet on imperfectly sampled data: EWSNet is robust to observational error. The model performance improves with an increase in the sequence length; the model performs well even for moderate infrequent sampling for both Dataset-W and Dataset-C. The infrequent temporal sampling is achieved by randomly selecting a subset of the original time series (increments of 0.01–1 of the actual length).

Detection of EWSs using classical trends in statistics such as autocorrelation can be highly sensitive to non-uniform sampling of data [18,19], with sporadic sampling increasing the probability of misidentifying catastrophic and non-catastrophic transitions, or failing to detect a transition at all. We investigated the robustness of the trained EWSNet models towards imperfectly sampled time series by re-sampling the time series. As expected, this imperfect sampling reduced the efficiency of generic EWSs. However, EWSNet models continued to provide robust predictions of approaching transitions; even when re-sampling retained only 40% of the original simulated data, the mean accuracy remained above 80% (figure 4).

### 3.2. Leave one model out cross validation

We performed a leave one model out cross validation experiment to analyse the extent of generalizability of the EWSNet. The EWSNet is trained using data from eight models each time and tested on the data from the left-out model. This results in a setting where the EWSNet is tested on data from a model exhibiting different nonlinearity compared with the training models. Six out of eight trained variants of EWSNet reliably predict the transitions in the test instances that are generated from a model of different origin (table 1). While the results indicate that EWSNet is reliable as an early warning indicator of time series with similar underlying traits as that of training data, there is further scope for improving the generalizability of the EWSNet through fine-tuning and training over other models and larger parameter ranges.

**Table 1.** Leave one model out cross validation experiment results: The EWSNet is trained and tested on each of the eight sets individually and the corresponding accuracies averaged over 25 trials are presented below (Note: we suspect the zero accuracy for sets 3 and 4 due to the low densities of the only lower state model used for training along with other saddle-node models with higher densities (shift from upper to lower state). However, originally when trained with both models, as in training with Dataset-W or Dataset-C, resolves this issue.)

Sl. No.	training data	test data	test accuracy over 25 trials
(1)	Model [2]–[9]	Model [1]	0.76 ± 0.05
(2)	Model [1], [3]–[9]	Model [2]	1.0 ± 0.0
(3)	Model [1]–[2], [4]–[9]	Model [3]	0.0 ± 0.0
(4)	Model [1]–[3],[5]–[9]	Model [4]	0.0 ± 0.0
(5)	Model [1]–[4], [6]–[9]	Model [5]	0.96 ± 0.04
(6)	Model [1]–[5], [7]–[9]	Model [6]	0.64 ± 0.12
(7)	Model [1]–[6], [8]–[9]	Model [7]	1.0 ± 0.0
(8)	Model [1]–[7],[9]	Model [8]	0.88 ± 0.09

### 3.3. Performance of ML models trained using generic EWSs

**Figure 5.**
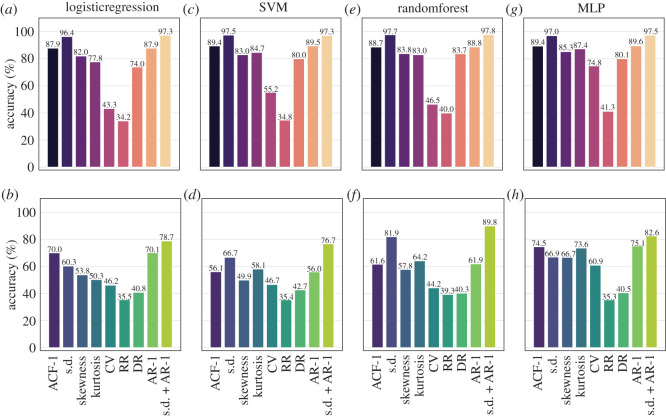
Performance of generic EWSs using (*a*,*b*) logistic regression, (*c*,*d*) support vector machine (SVM), (*e*,*f*) random forest, and (*g*,*h*) multilayer perceptron (MLP): The ML models are trained on EWSs extracted using various combinations of rolling window sizes and bandwidths. The results are presented as bar plots corresponding to each EWS indicator for Dataset-W (upper panel) and Dataset-C (lower panel). Among the best performing indicators are s.d., AR1 and s.d. + AR1. The s.d. + AR1 model improves the performance over the individual s.d. and AR1 models. The labels on the *x*-axis represent the generic EWSs, where ACF-1 represents autoregressive coefficient of AR-1 model, s.d. denotes standard deviation, CV represents coefficient of variation, RR is the return rate, DR denotes density ratio, AR-1 represents lag-1 autocorrelation and s.d. + AR1 represents composite EWS indicator consisting of s.d. and AR1.

While EWSNet is trained on the raw simulated time-series data, it is also possible to train ML models on the trends in generic EWSs calculated from these simulations and thus use ML in an attempt to detect similar trends in statistics such as autocorrelation in the validation datasets. To compare the efficacy of this approach to the EWSNet approach, we used four standard ML models [34,41,42] (logistic regression (LR), support vector machine (SVM), multilayer perceptron (MLP) and random forests (RF)) to characterize trends in generic EWSs in simulated time series. The input to the ML model is the concatenation of the generic EWSs captured for different combinations of bandwidths and window sizes (for details see electronic supplementary material, appendix, section S3). The results presented in figure 5 show that AR-1 and s.d. are the top two performing generic EWSs across both the datasets and across all the ML models, in accordance with prior literature [55]. Thus, we further trained ML models using a combination of AR-1 and s.d. which resulted in performance greater than the models trained on the individual EWSs (figure 5) and other pairwise combinations of EWSs (see electronic supplementary material, appendix, section S3, figures S3–S5).

Table 2 compares the accuracies of the generic EWSs-based ML models against the EWSNet for both Dataset-W and Dataset-C. EWSNet performs significantly better in distinguishing transitions and classifying time series for both the datasets: the *p*-value for the *t*-statistics is negligible, indicating that the null hypothesis of obtaining similar mean accuracy using classical ML method trained on generic EWSs as that of EWSNet should be rejected. Interestingly, irrespective of the ML model chosen—tipping points are more accurately identified in Dataset-W than Dataset-C. This can be explained by the short-scale fluctuations introduced by coloured noise, in Dataset-C, which can dampen the trend in the time series.

**Table 2.** Comparison of mean accuracy for various models: on comparing the mean accuracy, EWSNet appears to be the best performing model in classifying a critical transitional time series consistently for both the Dataset-W and Dataset-C. On passing the EWSs as input to the other ML models, the results are comparably close to the EWSNet for Dataset-W, but the accuracy declines for Dataset-C. The mean accuracy for the four classical ML models is comparable. The random forest is the best among them, with standard deviation and a combination of standard deviation and autocorrelation at lag-1 as features. The numeric value in ± denotes 95% confidence interval.

ML methods	EWSs	Dataset-W	Dataset-C
	s.d.	97.47 ± 0.09	66.69 ± 0.00
support vector machine	AR1	89.55 ± 0.17	55.95 ± 0.00
	s.d. + AR1	97.27 ± 0.14	76.70 ± 0.00
	s.d.	96.40 ± 0.14	60.27 ± 0.01
logistic regression	AR1	87.88 ± 0.22	70.08 ± 0.02
	s.d. + AR1	97.35 ± 0.11	78.74 ± 0.02
	s.d.	97.69 ± 0.12	81.93 ± 0.22
random forest	AR1	88.76 ± 0.20	61.94 ± 0.30
	s.d. + AR1	97.77 ± 0.16	89.79 ± 0.82
	s.d.	97.02 ± 0.69	66.94 ± 1.55
multilayer perceptron	AR1	89.58 ± 0.45	75.10 ± 0.82
	s.d. + AR1	97.47 ± 0.16	82.56 ± 2.10
EWSNet		99.46 ± 0.01	95.93 ± 0.02

Populations experiencing red noise are relatively more threatened and found to encounter long periods of no-survival conditions [45,56] and their transitions need to be anticipated. Therefore, we compared the EWSNet against the ML models on Dataset-C (the test set contains time series with high correlated noise). The metrics accuracy, area under the curve (AUC), true positive rate (TPR), precision and F-score for each of the models are presented in electronic supplementary material, appendix, section S3, figure S6), again EWSNet outperforms other methods for these red noise time series.

### 3.4. Applicability and robustness of EWSNet to real-world and experimental data

**Figure 6.**
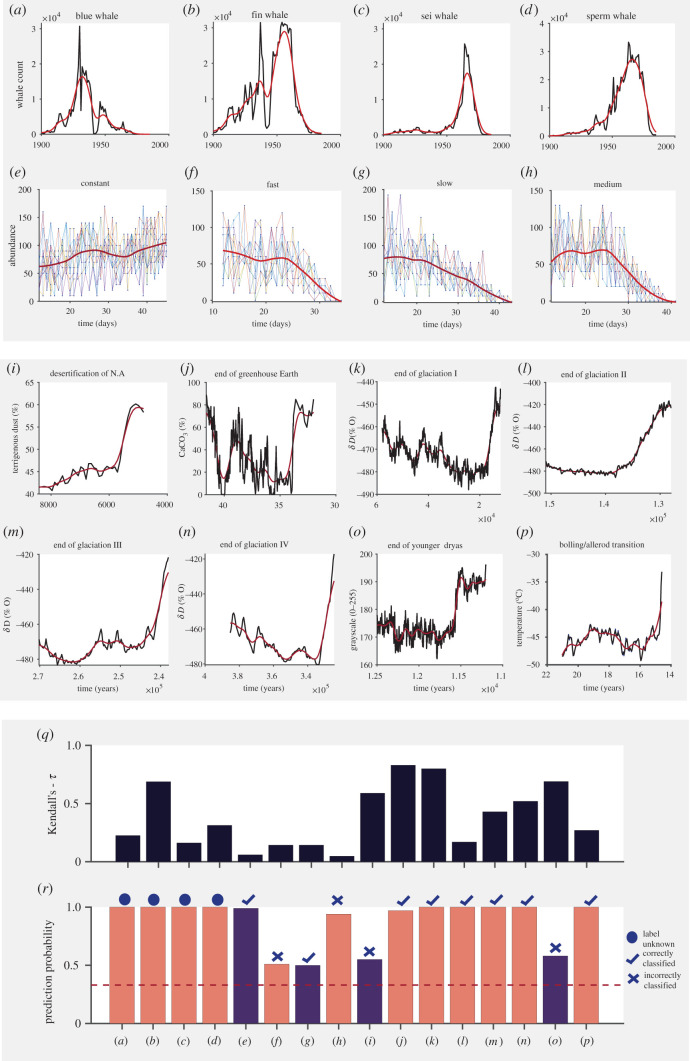
(*Overleaf*.) Performance on real-world and experimental time series: time series data of: (*a*–*d*) number of whales caught per year, (*e*–*h*) abundance of *Didinium nasutum* populations that were exposed to four different experimental treatments and (*i*–*p*) climate systems. In (*a*–*p*), the red curve is the time series trend. Histograms showing performance of (*q*) AR-1 using Kendall’s-*τ*, and (*r*) the EWSNet using prediction probability, respectively, for the time-series data (*a*–*p*). In (*r*), the red dashed line denotes prediction probability due to chance (the confidence with which the EWSNet classifies the transition in time series, for a three-class classification problem this stands at 33.33%). The results in (*q*–*r*) are presented for data considered up till tipping as shown by the grey solid line (see electronic supplementary material, appendix, figures S7 and S8). The results for dryland ecosystem are presented in electronic supplementary material, appendix, table S3 and figure S9. Colour peach (purple) denotes the detected type of transition by EWSNet—catastrophic (non-catastrophic).

The noisy nature of non-simulated data means that the effectiveness of EWSs is often variable [57]. To test the reliability of EWSNet when predicting real-world data, we tested it on 17 published palaeoclimatic [5] and ecological datasets [21,22,43] (see electronic supplementary material, appendix, section S4, figures S7 and S8). We used EWSNet trained with simulated data to ascertain whether the critical transitions exhibited by these time series were predictable. Using a classical ML model approach in such real-world time series (which vary in length) is challenging and requires steps to make their lengths equal to those of the training data. These additional steps will either pad, interpolate or truncate the sequences, potentially leading to information loss and erroneous results. By contrast, EWSNet can classify time series of varying lengths due to the global average pooling post-convolution layers, making the model dynamic. We present the prediction probabilities for the EWSNet, and Kendall’s-*τ* correlation coefficient for the generic EWS (AR-1) (see electronic supplementary material, appendix, table S3). The EWSNet classifies transitions in 9 out of 13 real-world time series (for which the ground truth label is known *a priori*) over 25 trials (figure 6). EWSNet results in the average prediction probability greater than or equal to 95% for all the correctly classified series except *Didinium nasutum* (slow) for which the prediction probability continues to remain close to 50% even up until the tipping. The ground truth labels corresponding to the real-time series depicted in figure 6*a*–*d* are unknown, though EWSNet classifies them as catastrophic transitions. This result represents the first application of real-world time series analysed using deep learning models trained only on simulated time series.

## 4. Discussion

Previously developed EWSs [12,21,23] have had mixed success in predicting approaching transitions, particularly in noisy real-world data. Here, we take an entirely new approach to predicting transitions in complex systems by developing a deep learning model (EWSNet) framework that can forecast not only an approaching tipping point but also discern whether it is catastrophic or not. The key characteristic of our model is its non-dependence on *a priori* statistical features such as increasing autocorrelation in a time series.

Key to the applicability of warning signals approaches to real-world conservation issues is their ability to make predictions in the face of incomplete and noisy time series [19,58]. EWSNet is likely to provide reliable predictions on test time series following critical behaviour similar to that of the training set. Having prior knowledge (correlated or uncorrelated noise, cyclicity or non-cyclicity) about the property of the time series will definitely help in training an appropriate ML model. However, in the absence of this knowledge, a feasible solution would be to train EWSNet on data from ‘all possible’ scenarios of coloured and white noise. Our work follows the second direction—train EWSNet using data from all the well-studied critical behaviour models. EWSNet performs reasonably well when presented with only 5% of randomly sampled data while performance improves steadily with the availability of more information on the time series (figure 4). While imperfect sampling can add uncertainty in the value of each point in a time series (and has been shown to have detrimental affects to CSD-based warning signals [19]), time-series data can also be limited temporally. Consequently, understanding what section of a time series provides the most predictive features for EWSNet is important for it to be applied to real-world data. Our results suggest that EWSNet performs significantly better when presented with time-series data closer to the tipping point, rather than from further away (see electronic supplementary material, appendix, figure S2), a pattern also observed in CSD and trait-based warning signals [22]. We speculate EWSNet classifies transitions learning from the higher-order nonlinearities present in the time series apart from system’s resilience. Nevertheless, understanding the learning strategy of the EWSNet can be engrossing yet a highly challenging problem, as is explaining the behaviour of any deep neural network model.

However, data quality remains a key issue determining the reliability of predictive tools, a point exemplified by the whaling data analysed within this paper (figure 6*a*–*d*). EWSNet predicts that whale populations exhibit a catastrophic bifurcation, probably driven by the rapid change in the number of whales caught each year alongside signals embedded within the time-series data (figure 6). The number of whales harvested each year is, however, a function not only of the size of the whale population but also the harvesting effort and so, while EWSNet correctly predicts the collapse of whale populations previously identified [22], whether the classification of a catastrophic tipping generated by EWSNet is correct remains unknown, as thus far no work has explicitly classified the tipping points observed in these data. Thus, EWSNet appears to robustly predict tipping points in the face of such noisy real-world data, data quality will determine both how far in advance such predictions are reliably made, and whether the classifications generated are correct.

EWSNet’s ability to discriminate between catastrophic and non-catastrophic transitions sets it apart in the field of early warning indicators, where existing techniques have only been able to ascertain whether a system is close to a transition, but not the type of transition [14,51]. This has significant implications for the management of complex systems, as some transitions (particularly catastrophic transitions which are typified by sudden changes in the state of a system) are hard to reverse and can lead to loss of function. Examples such as the shift to algal-dominated states in fresh waters, where cyanobacteria often proliferate [59], highlight this need to predict such rapid nonlinear shifts, as reversing them can be difficult [10]. Consequently, the ability to infer whether a system may exhibit an abrupt (hard to reverse) or smooth (easier to reverse) transition would allow rapid prioritization of which systems to target for further study [60].

Although our research takes a novel approach to the detection of early warning indicators, in its current form EWSNet does not constitute a universal indicator of tipping points. However, one of the key advantages of a deep neural network approach is that—unlike the approach of classical CSD-based warning signal—it can be trained in an unbiased way making few *a priori* assumptions about the dynamics of the system in the region of a tipping point. For example, although, we have analysed eight real-world atmospheric empirical datasets, the EWSNet is trained using only one atmospheric model (electronic supplementary material, appendix, Model [5] in table S1). EWSNet when tested on climate data collected from different sources, as in Dakos *et al.* [5], classifies six out of these eight climate time-series data correctly with high prediction probability, suggesting that this approach has considerable merit. Alternative approaches—such as fitting the various models to the empirical data and assessing which best describes the observed dynamics or machine learning techniques applied to CSD-based indicators—offer other potential avenues of study. However, model fitting approaches make more assumptions about the underlying structure of the system (i.e. that the dynamics of the system are well represented by one of the models). EWSNet avoids these assumptions by leveraging against the predictions of theory which suggest that, in the vicinity of a critical point, any nonlinear model exhibiting a particular bifurcation (say a saddle-node bifurcation) is representative of all saddle-node bifurcations [61]—the same rationale for the detection of classical CSD-based indicators. Moreover, EWSNet outperforms ML approaches fitted to CSD-based indicators (table 2).

In conclusion, EWSNet serves as a framework which has the potential to reliably predict transitions in a broad suite of simulated and empirical systems. We believe that the EWSNet captures features indicative of approaching transitions and characteristics, the generic CSD-based EWSs do not capture. Moreover, classical approaches using generic EWSs require a careful selection of suitable bandwidth, and window size [18,62], both of which EWSNet is robust to. A recent study by Bury *et al.* [63] take a step forward by classifying bifurcations using deep learning models. Future work could include retraining EWSNet to classify bifurcations and develop machine learning models to further predict not just if, but when a critical transition will occur. Moreover, when trained using data across a large array of critical behaviours (e.g. global and higher co-dimension bifurcations, bifurcation without critical slowing down, and sharp transition without bifurcation, etc.), will open up the possibility of real-time monitoring of many real-world systems such as global climate, ecosystems and cell dynamics with a negligible computational cost. EWSNet is not designed to replace the in-depth study and understanding of a system. Rather, EWSNet offers a first-pass tool to prioritize at-risk systems for further study, but which can be expended upon with further model training to provide an increasingly robust and widely applicable framework into the future. Nevertheless, there is always further scope for increasing generality and robustness of EWSNet through fine-tuning and training over different labels (different types of transitions) using different mathematical models with larger parameter ranges.

## Data accessibility

Codes and data are available in a Zenodo repository (http://doi.org/10.5281/zenodo.5027683). The data are provided in electronic supplementary material [64].
